# Development and validation of prognostic survival models in newly diagnosed Parkinson's disease

**DOI:** 10.1002/mds.27177

**Published:** 2017-10-04

**Authors:** Angus D. Macleod, Ingvild Dalen, Ole‐Bjørn Tysnes, Jan Petter Larsen, Carl E. Counsell

**Affiliations:** ^1^ Division of Applied Health Sciences University of Aberdeen Foresterhill Aberdeen UK; ^2^ Department of Research, Section of Biostatistics Stavanger University Hospital Stavanger Norway; ^3^ The Norwegian Centre for Movement Disorders Stavanger University Hospital Stavanger Norway; ^4^ Institute of Clinical Medicine University of Bergen Bergen Norway; ^5^ Department of Neurology Haukeland University Hospital Bergen Norway; ^6^ Network for Medical Sciences University of Stavanger Stavanger Norway

**Keywords:** Parkinson's disease, prognosis, mortality, dependency

## Abstract

**Objective:**

The objective of this study was to develop valid prognostic models to predict mortality, dependency, and “death or dependency” for use in newly diagnosed Parkinson's disease (PD).

**Methods:**

The models were developed in the Parkinsonism Incidence in North‐East Scotland study (UK, 198 patients) and validated in the ParkWest study (Norway, 192 patients), cohorts that attempted to identify and follow‐up all new PD cases in the study area. Dependency was defined using the Schwab & England scale. We selected variables measured at time of diagnosis to include in the models. Internal validation and external validation were performed by calculating C‐statistics (discrimination) and plotting observed versus predicted risk in quantiles of predicted risk (calibration).

**Results:**

Older age, male sex, increased severity of axial features, and Charlson comorbidity index were independent prognostic factors in the mortality model. Increasing age, higher smoking history, increased severity of axial features, and lower MMSE score were independent predictors in the models of dependency and “death or dependency.” Each model had very good internal calibration and very good or good discrimination (internal and external C‐statistics for the models were 0.73–0.75 and 0.68–0.78, respectively). Although each model clearly separated patients into groups according to risk, they tended to overestimate risk in ParkWest. The models were recalibrated to the baseline risk in the ParkWest study and then calibrated well in this cohort.

**Conclusions:**

We have developed prognostic models for predicting medium‐term risk of important clinical outcomes in newly diagnosed PD. These models have validity for use for stratification of randomization, confounder adjustment, and case‐mix correction, but they are inadequate for individualized prognostication. © 2017. The Authors. Movement Disorders published by Wiley Periodicals, Inc. on behalf of International Parkinson and Movement Disorder Society.

Parkinson's disease (PD) is a progressive disabling disorder with higher mortality, disability, and dementia than in controls.^1^, ^2^ Being able to predict outcome has many benefits, including individualized risk prediction with improved information for patients, stratifying or personalizing treatments according to prognosis, improving design of clinical trials (eg, selection of participants, stratifying randomisation, or adjustment for covariates), and adjusting for case‐mix when comparing different cohorts.^3^, ^4^ It is important that appropriate methods are used to develop prognostic models, including a representative patient sample and adequate external validation to demonstrate that the model can be used in settings other than those in which it was developed.^3^, ^4^

Only one externally validated prognostic model has been published for use in PD, but it is limited to predictions at a single timepoint (at 5 years) and uses a single composite outcome.^5^ No prognostic models have been published for specific outcomes in PD. We therefore aimed to (1) develop prognostic models for predicting 3 important outcomes over time in PD, that is, mortality, dependency (needing help with basic activities of daily living such as walking, dressing, toileting), and the composite outcome of “death or dependency” (which may be a useful simple clinical measure of disease progression)^6^ and (2) to validate these models in an international cohort. We have previously published data on individual predictors of dependency and “death or dependency” in PD,^7^ but this paper extends this work significantly by combining prognostic factors to develop and validate predictive models for these outcomes and mortality.

## Materials and Methods

We developed the prognostic models in the Parkinsonism Incidence in North‐East Scotland (PINE) study and performed external validation in the ParkWest study in prospective, population‐based incident cohorts of PD (ie, which attempted to recruit and follow‐up all new PD cases in the specified time period and area).

### PINE Study (Development Cohort)

The PINE study was recruited during the following 2 incidence periods: an 18‐month pilot phase in area with population of 148,600 beginning November 2002 and the 36‐month main study phase in population of 317,357 people beginning April 2006.^8^, ^9^, ^10^ Patients were recruited using multiple, overlapping strategies for case ascertainment, including writing to general practitioners and hospital specialists asking them to refer suspected cases, hand‐searching neurology/geriatrics referral letters, and electronic searching of general practice and hospital discharge databases. All those referred or identified through the searches and who did not have a previous diagnosis of a parkinsonian disorder were invited to be assessed by a neurologist with a special interest in movement disorders (or supervised trainee). Those with parkinsonism (defined as 2 or more of rest tremor, bradykinesia, rigidity, or unexplained postural instability) were invited to consent to long‐term annual follow‐up. Consenting patients were reviewed annually with a range of clinical assessments. Only those diagnosed with idiopathic PD (guided by the UK PD Society Brain Bank criteria)^11^ were included in this analysis.

### ParkWest Study (Validation Cohort)

The ParkWest study sought to recruit all new PD cases over 22 months from November 1, 2004, in 4 Norwegian counties comprising a population of 1,052,075.^12^ Similar to the PINE study, multiple, overlapping strategies were used for case ascertainment, including hand‐searching referral letters, notification of general practitioners and relevant hospital specialists asking them to refer suspected cases, and electronic searching of hospital databases and general practitioners' electronic medical records systems. All new cases were invited to consent to follow‐up with twice‐yearly assessment, including a range of clinical assessments. PD diagnoses were made using the UK PD Society Brain Bank criteria^11^ and the Gelb criteria.^13^

Both studies were approved by appropriate ethics committees (Multi‐centre Research Ethics Committee for Scotland, Edinburgh, UK, University of Bergen, Bergen, Norway) and were conducted with the informed consent of the patients involved.

#### Outcome Definition

Data on mortality were derived from notifications by relatives/general practitioners plus surveillance by the UK national death registers in the PINE study. Functional dependency was measured by the Schwab & England scale^14^ at follow‐up visits and defined using a cut‐off of < 80% (80% = completely independent in most chores; 70% = not completely independent). The word *chores* was consistently interpreted as basic activities of daily living (walking, personal hygiene, dressing, toileting, feeding) by both study teams. The Schwab & England scale has been partly validated for use in PD as a measure of activities of daily living,^15^, ^16^, ^17^, ^18^ but its validity as a dichotomous measure of dependency/independency has not been established. Nevertheless, it does have face validity for this purpose.

#### Predictor Variables

We selected potential baseline predictors (ie, measured at diagnosis) to include in the model based on previously reported associations,^1^, ^19^ a previous analysis of the measures of motor impairment with best predictive value in the PINE study (in which measures of axial severity were better predictors of long‐term outcomes than other measures of motor function, including the postural‐instability‐gait‐difficulties/tremor‐dominant classification),^20^ and clinical knowledge. We limited the number of candidate predictors to avoid overfitting.^21^ We included age in the models irrespective of statistical significance because, on the basis of previous studies and clinical knowledge, it strongly predicts many outcomes in PD. The other candidate baseline variables were selected using a backward stepwise selection process (see Model Development): sex, pack years of smoking history, Charlson comorbidity score,^22^ severity of bradykinesia (sum of bradykinesia items from UPDRS [part III] motor score), severity of axial features (sum of speech, facial expression, face tremor, neck rigidity, arising from chair, posture, gait, and postural instability items from the UPDRS [part III] motor score), mini‐mental state examination (MMSE) score, and Hoehn & Yahr stage. We previously compared the Charlson comorbidity score with 2 other scales and found it was more reliable and had better predictive validity.^23^ There were no missing data for predictor variables in either study. Treatment‐related variables were not included to allow the models to be used at time of diagnosis (ie, before treatment initiation) and in trials of de novo treatment in PD.

#### Model Development

We developed 3 models to predict time to all‐cause mortality, time to dependency, and time to “death or dependency.” We used Weibull proportional hazards parametric survival modelling to allow direct estimation of the baseline hazard function (the hazard function when centred covariates are set to 0), for ease of out‐of‐sample prediction and for straightforward validation. We investigated other parametric distributions of the baseline hazard, but none provided better fit adjusted for complexity (assessed by the Bayesian information criterion). Univariable analysis was performed with each candidate predictor in turn, and then those variables with association with the outcome of interest (*P* < .2) were included in a backward stepwise regression model. A probability cut‐off of .1 was used for removal of variables from the model (other than age). We checked whether the proportional odds or probit scale fitted better than the proportional hazards scale, but they did not. Functional form was tested by assessing whether a 2‐power fractional polynomial (ie, nonlinear terms) provided better fit.^24^ The influence of individual observations on the models was assessed by calculating the likelihood displacement.^25^ Because the effect of baseline Charlson score on mortality varied with time from the baseline assessment, an interaction between Charlson score and 2 discrete periods of time was added to the model (with approximately equal numbers of deaths in each time period), resulting in significantly improved fit (*P* = .001). The significance of interactions between age and other variables in the model were assessed, but other interactions were not tested because of limited power. Shrinkage was not applied during prognostic modelling development as the shrinkage factors were all >0.85.^26^

For the dependency model, patients who were dependent at time of diagnosis were excluded and patients who died or were lost to follow‐up prior to becoming dependent were censored at the time they were last seen. We assumed that censoring because of death was independent of dependency because most deaths in those who were not previously known to be dependent were not a result of PD and therefore unlikely to be related to dependency. Patients remaining alive and independent were censored at the time of their last visit (or up until January 19, 2015 for surviving patients in the PINE study). Two patients were excluded from the development of the mortality model and 1 from the dependency model because of unusual outlying values together with high influence on the model.

#### Model Validation

We assessed validation by measuring discrimination (the ability of a model to distinguish different risks between individuals) and calibration (the degree of agreement between observed event probability and predicted event probability in groups of patients defined by the prognostic index [*xβ*]). Discrimination was measured using the C‐statistic, the proportion of all possible pairs of observations in the dataset correctly ranked in terms of risk.^27^ Calibration was assessed by plotting observed versus predicted survival probabilities in 4 quantiles of predicted risk derived from the models (ie, the prognostic index [*xβ*]). First, apparent discrimination and calibration was assessed, that is, the direct performance of the models in the PINE dataset in which they were created. Second, (so‐called) optimism‐adjusted estimates of discrimination in the PINE dataset were assessed using 500 bootstrapped samples for each model.^3^, ^28^ Third, the models developed in the PINE study were externally validated by measuring their discrimination and assessing their calibration in the ParkWest study.^3^, ^28^

#### Model Recalibration

The models were recalibrated to account for a different baseline risk in the ParkWest study.^29^ This was done by iterating the constant and shape parameter of the model so that the curve of mean predicted survival probabilities for all patients in the ParkWest study was a close fit to the observed Kaplan‐Meier survival probabilities (leaving the other parameter estimates unchanged). The calibration plots were repeated using the new constant and shape parameter to derive the predicted values.

All statistical analyses were performed with Stata version 12.1 (StataCorp LP, College Station, Texas). Appropriate reporting guidelines have been followed.^30^

## Results

Supplementary Figure 1 shows recruitment to the PINE and ParkWest studies. Consent to follow‐up was higher in the PINE study than in the ParkWest study (94% vs 81%). A total of 198 patients with PD in PINE and 192 patients with PD in ParkWest were included in the analyses of mortality. Of the patients, 22 (11%) were excluded from analyses of dependency in the PINE study and 30 in the ParkWest study because they were already dependent at baseline (16%). The patients in the PINE study were older (mean age at diagnosis 72.5 vs 67.9), had shorter median symptoms duration at baseline assessment (13 vs 20 months), higher smoking history, slightly higher disease impairment and disease stage, and more comorbidities (Table 1). Follow‐up data were available for between 6 and 12 years from diagnosis in the PINE study and between 6 and 8 years from diagnosis in the ParkWest study.

**Table 1:** Baseline characteristics

Baseline variable	PINE, N = 198	ParkWest, N = 192
Mean age in years at diagnosis (SD)	72.5 (10.4)	67.9 (9.3)
Number male (%)	119 (60)	117 (61)
Median symptom duration in months (IQR)	13 (9‐24)	20.0 (13.6‐36.8)
Mean H&Y stage (SD)	2.3 (0.8)	1.9 (0.6)
Mean UPDRS motor score (SD)	25.1 (11.6)	23.5 (11.2)
Mean MMSE (SD)	28.1 (2.3)	27.8 (2.5)
Median Charlson score (IQR)	1 (0‐2)	0 (0‐1)
Median pack years of smoking history (IQR)	0 (0‐15)	8 (3‐20)

IQR, interquartile range; PINE, Parkinsonism Incidence in North‐East Scotland; SD, standard deviation.

### Mortality

**Figure 1:**
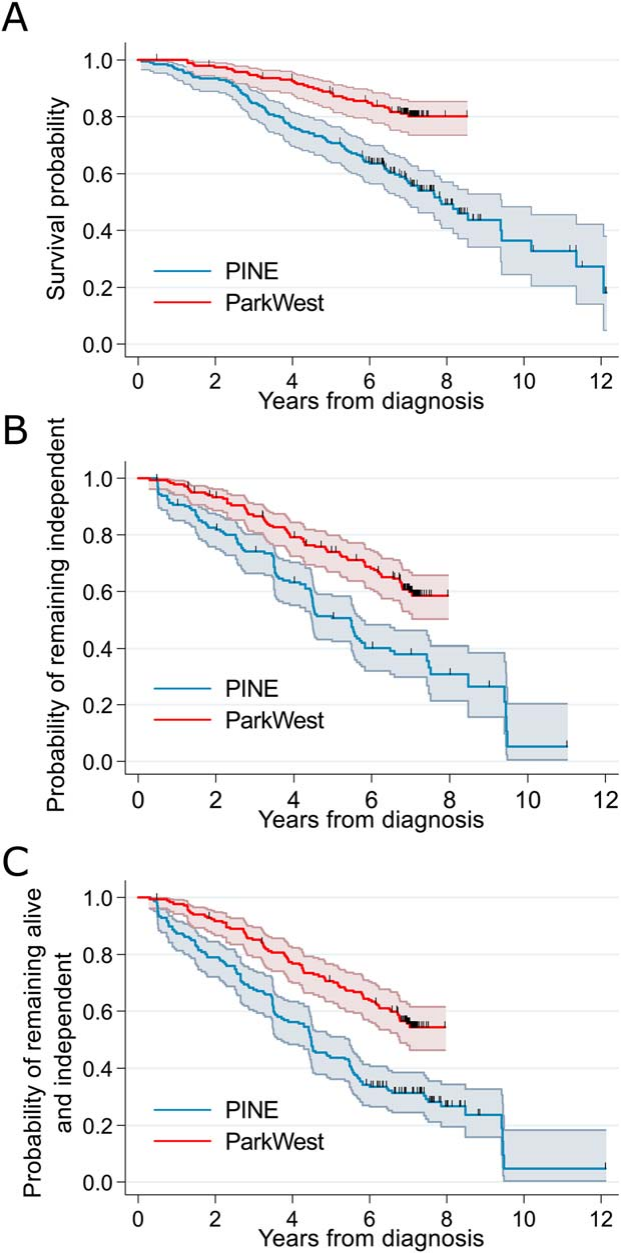
Plots of survival in the Parkinsonism Incidence in North‐East Scotland (PINE) and ParkWest studies. Plots of Kaplan‐Meier probability of (**A**) survival, (**B**) remaining independent (with deaths censored), and (**C**) remaining alive and independent. Colored bands represent 95% confidence bands for the Kaplan‐Meier survival probabilities. Vertical marks represent censored observations. [Color figure can be viewed at wileyonlinelibrary.com]

**Figure 2:**
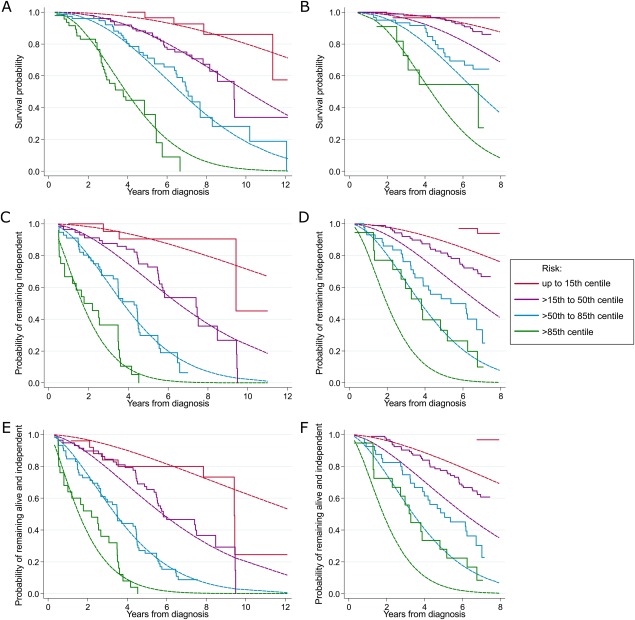
Internal and external calibration plots. Plots of model calibration showing the observed and predicted probabilities of outcome in quantiles of predicted risk of survival in the Parkinsonism Incidence in North‐East Scotland (PINE) study (**A**) and in the ParkWest study (**B**), of remaining independent in the PINE study (**C**) and in the ParkWest study (**D**), and of remaining alive and independent in the PINE study (**E**) and in the ParkWest study (**F**). The dashed lines indicate the probabilities predicted by the model, and the solid lines indicate the Kaplan‐Meier survival function. [Color figure can be viewed at wileyonlinelibrary.com]

**Figure 3:**
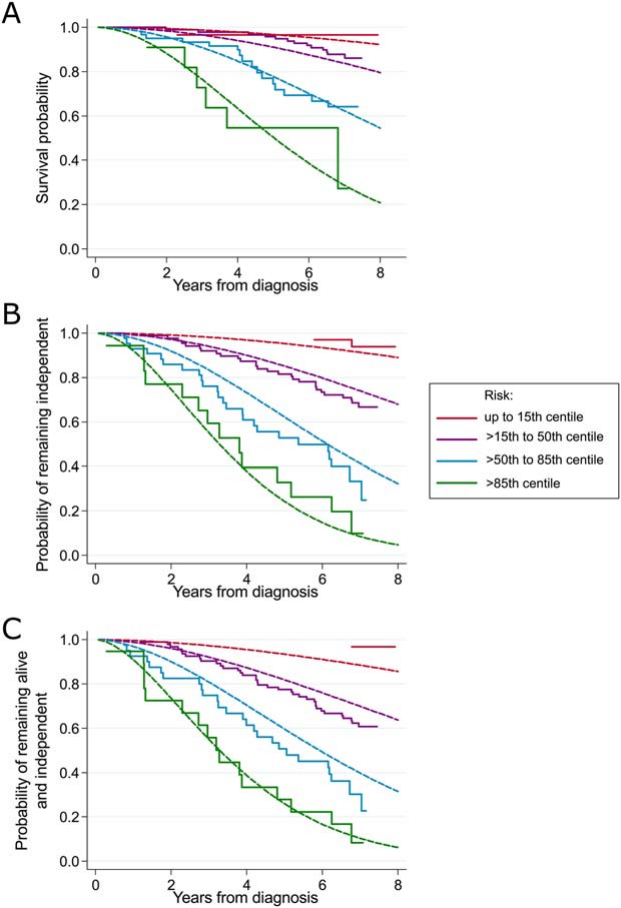
Recalibrated model calibration plots. Plots of model calibration showing the observed and predicted probabilities of outcome in quantiles of predicted risk in the models recalibrated for the baseline risk in the ParkWest study: (**A**) survival, (**B**) dependency, and (**C**) death or dependency. The dashed lines indicate the probabilities predicted by the model and the solid lines indicate the Kaplan‐Meier survival function. [Color figure can be viewed at wileyonlinelibrary.com]

Mortality was higher in the PINE study than in the ParkWest study (see Fig. 1A). We developed 2 models predicting mortality, with and without the Charlson comorbidity index, to enable use in settings where comorbidity data is not available. Age, sex, and severity of axial features were included in both models (Table 2). The model increased mortality with increasing comorbidity burden in the first 4 years of follow‐up and a nonsignificant decrease in mortality with increasing baseline Charlson score after 4 years follow‐up. Apparent discrimination and calibration were very good (C‐statistics 0.75 and 0.73 for model with and without Charlson score, respectively; see calibration plot in Fig. 2A). Calibration plots for the model without Charlson score are not shown, but are almost identical. Both models discriminated better in the external cohort than internally (C‐statistics 0.76 and 0.78 for models with and without comorbidity burden). However, although the calibration plot in the ParkWest study showed good separation into groups according to prognostic risk, the model tended to overestimate risk of death except in the highest‐risk quantile (Fig. 2B). Using the recalibrated model, calibration was very good (Fig. 3A).

**Table 2:** Prognostic models

			C‐statistic (95% CI) – model discrimination		
			Internal (PINE)		
Outcome	Baseline prognostic factor	Hazard ratio (95% confidence interval)	Apparent	Optimism corrected	External (ParkWest)
All‐cause mortality	Age (10‐year increase)	2.02 (1.51‐2.71)	0.75 (0.70‐0.80)	0.73	0.76 (0.68‐0.85)
	Male sex	1.78 (1.13‐2.82)			
	Axial severity (5‐point increase)	1.33 (1.08‐1.66)			
	Charlson score (effect in first 4 years of follow‐up)	1.31 (1.12‐1.52)			
	Charlson score (effect after first 4 years)	0.83 (0.63‐1.08)			
	Shape parameter	2.28 (1.90‐2.74)			
	Constant	−2.66 (−3.09 to 2.23)			
All‐cause mortality (alternative model excluding Charlson score)	Age (10‐year increase)	2.12 (1.58‐2.84)	0.73 (0.68‐0.78)	0.71	0.78 (0.70‐0.86)
	Male sex	1.85 (1.17‐2.91)			
	Axial severity (5‐point increase)	1.41 (1.13‐1.76)			
	Shape parameter	1.92 (1.61‐2.28)			
	Constant	−2.38 (−2.77 to 2.00)			
Functional dependency	Age (10‐year increase)	2.15 (1.61‐2.86)	0.77 (0.72‐0.82)	0.74	0.68 (0.61‐0.75)
	Smoking history (10‐pack‐year increase)	1.15 (1.05‐1.27)			
	Axial severity (5‐point increase)	1.74 (1.28‐2.35)			
	MMSE score	0.86 (0.77‐0.96)			
	Shape parameter	1.85 (1.56‐2.18)			
	Constant	1.15 (0.05‐28.1)			
Death or dependency	Age (10‐year increase)	1.91 (1.50‐2.44)	0.74 (0.69‐0.79)	0.73	0.68 (0.62‐0.75)
	Smoking history (10‐pack‐year increase)	1.16 (1.07‐1.26)			
	Axial severity (5‐point increase)	1.56 (1.18‐2.05)			
	MMSE score	0.86 (0.78‐0.95)			
	Shape parameter	1.66 (1.43‐1.92)			
	Constant	1.89 (0.11‐32.8)			

### Dependency

The probabilities of developing sustained dependency over time were higher in the PINE study than in the ParkWest study (Fig. 1B). Median time until dependency was 5.5 years in the PINE study, but was not reached in the ParkWest study. The model of dependency developed in PINE included age, smoking history, severity of axial features, and MMSE score (Table 2). This model had relatively high apparent discrimination (C‐statistic 0.77) and very good calibration (Fig. 2C). Discriminative performance was also very good in the validation cohort (C‐statistic 0.75), and although the calibration plots showed clear separation by prognostic risk, there was systematic overestimation of risk of dependency in the validation cohort (Fig. 2D). However, the recalibration plots show much better calibration (Fig. 3B).

### Death or Dependency

The risk of becoming “dead or dependent” was substantially higher in the PINE study than in the ParkWest study during most of the follow‐up period (see Fig. 1C). The model of “death or dependency” developed in PINE included the same variables as in the model of dependency (Table 2). This model had good apparent discrimination (C‐statistic 0.74) and very good calibration (Fig. 2E). The model discriminated slightly better in the ParkWest study (C‐statistic 0.76). The calibration plots showed clear separation by prognostic risk, but there was systematic overestimation of risk of “death or dependency” in the validation cohort (Fig. 2F). The recalibrated model showed much improved calibration (Fig. 3C).

For each model, assumptions were satisfied, linear form was appropriate for interval variables, and there was no evidence for interactions between age and other variables. Supplementary Appendix 1 contains a risk prediction calculator.

## Discussion

These 3 prognostic models combine clinical features at the time of diagnosis to predict important clinical outcomes in PD. They performed well in the PINE cohort and when tested in the ParkWest cohort, they had very good discrimination. Although there was good separation into prognostic groups, there was a systematic overestimation of risk in the patients in the ParkWest study. We developed recalibrated models that had good discrimination and calibration in the Norwegian cohort.

These models are novel. Although many previous studies have studied prognostic factors for mortality and dependency in PD,^1^, ^19^ and another study has developed, but not externally validated, a prognostic model for several outcomes including mortality,^31^ we are unaware of any externally validated prognostic model that can be used to calculate risk of specific outcomes in PD. One recent study has published a prognostic model developed in the Netherlands, and validated in England, which combined 3 variables (age, severity of axial features, and verbal fluency) to predict a composite outcome (postural instability, dementia, or death) at 5 years.^5^ Although this model discriminated well (C‐statistic 0.77 in the development cohort and 0.85 in the validation cohort) and calibrated well, its use is limited to predictions at a single timepoint and the components of the composite outcome are not equal in severity. Our models have the distinct advantages of predicting risk at multiple timepoints and of predicting specific outcomes.

We do not here discuss particular prognostic factors for dependency outcomes as we have done so previously.^7^ The finding that comorbidity was associated with mortality but not the dependency outcomes may be because many deaths, particularly early deaths, were related to comorbid disease, whereas most disability was related to PD itself. The interaction between comorbidity burden and time suggests that baseline comorbidity is more important for early mortality than for later mortality (given survival beyond year 4).

This study has 3 principal strengths. The first lies in the study designs, which both aimed to reduce selection bias by attempting to include all patients in the population with newly diagnosed PD. However, because PD is predominantly a disease of the elderly, there were relatively few young‐onset cases studied (8.5% < 55 at diagnosis). Although we are unaware of evidence that the effects of the prognostic factors are modified by age, the models should be used cautiously in young‐onset patient groups until they have undergone further validation in these groups.

The second main strength is the international external validation of the models. The key threat to model validity is overfitting as the model may fit too closely the idiosyncrasies of the development dataset resulting in overoptimistic estimates of model performance.^3^ Demonstrating that the model discriminates well in another cohort provides direct evidence of its generalizability to other settings and utility for certain applications. The third strength is that the models were developed following recommendations for prognostic studies^4^ using appropriate statistical methods.

An important limitation of this study relates to sample size. Although with about 100 deaths in the development cohort about 10 predictors can be studied without excessive overfitting,^21^ this limited selection of candidate predictors and the investigation of potential interactions in the models. Therefore further work in larger studies or combining studies could result in better models for individualized risk prediction.

A second important limitation is poor calibration of the models in the external cohort, which is probably mainly attributable to the higher overall risk of mortality and dependency in the PINE study. This must be related to factors which were not included (and therefore adjusted for) in the models. Differences in mortality are most likely explained by differences in non‐PD‐related mortality as it is unlikely the pathogenesis of PD is fundamentally different between the 2 countries and the burden of the diseases that most commonly lead to death (cardiovascular disease, respiratory disease, and malignancy) is higher in the United Kingdom than in Norway,^32^ which is reflected in the higher mortality rates in the United Kingdom than in Norway.^33^ Although there are no comparative population data on dependency available, the comorbidity rate was higher in the PINE study than in the ParkWest study, which may have led to higher dependency risk in the PINE study. Other possible explanations for these differences include the older average age of PINE participants and the lower consent rate in the ParkWest cohort if frailer patients were less likely to consent.

Because we have demonstrated the external validity of these models in terms of discrimination, we anticipate that these models will have several uses as research tools that separate patients into groups according to prognostic risk. These include in clinical trials design (eg, selection of participants for inclusion based on baseline predicted prognosis, stratification of randomization, and adjustment of analyses), adjustment for confounding in observational studies, and adjustment for case‐mix (eg, in comparing cohorts from different studies, countries, hospitals).^3^ These models combine simple and easy‐to‐collect variables that can easily be gathered in the clinic or in a research context.

Similarly, these models could also be used in clinical practice for stratification of treatment according to prognostic risk. Although there is no definite indication for this at present, this may be useful if disease modifying treatments with potentially serious side effects become available for PD. For example, they could be used to select people with the worst prognosis to try such treatments. However, these models are only a starting point; before these models can be used for individualized or personalized medicine, we need (1) further validation; (2) improved prediction, for example, adding biomarkers such as genetic or imaging factors; (3) simple implementation for use in clinical practice; and (4) evaluation of benefit versus harm, ideally in randomized trials^34^, ^35^ because the use of models to guide treatments may cause harm as well as benefit (eg, from false negative or false positive predictions). It will be important to develop flexible dynamic models that use accumulating data over time (such as disease progression, motor or nonmotor complications) to predict later outcomes but clearly those data are not available at diagnosis, and therefore models that use baseline data only are relevant for use in early disease.

Although prognostic models that discriminate well can be useful for research purposes and for treatment stratification even if they do not calibrate well, individualized risk prediction requires better precision than these uses and must calibrate well in the settings in which they are used. Although the original models worked well in North‐East Scotland, it was necessary to recalibrate to the ParkWest population in Western Norway. Therefore, the models should be recalibrated before use for individualized risk prediction in other geographical areas. This can be easily done if estimates of survival rate or progression to dependency are available.

In conclusion, we have developed and validated prognostic models to predict important outcomes in PD that use predictors that can be easily measured in the clinic setting. We have demonstrated that these models have sufficient validity to be used in a research context, but that recalibration is required before use in other geographical areas for individualized risk prediction. Further work includes (1) developing more‐accurate models with individual‐patient data meta‐analyses of representative studies; (2) further validation in a young‐onset cohort; (3) further simplification for use in case‐mix correction (eg, to identify which components of the axial features variable need to be collected); (3) updating these models to include biomarker data, for example, genetic, imaging, or biochemical data; (4) updating baseline risk in the models in the future if, for example, disease‐modifying treatments are introduced, which would alter the natural history of the disease; and (5) evaluation of their use in clinical management of individual patients. The use of these models in randomized controlled trials to stratify randomization by baseline prognosis would allow further validation by comparing participants' predicted and actual outcome.

## Author Roles

1) Research project: A. Conception, B. Organization, C. Execution; 2) Statistical Analysis: A. Design, B. Execution, C. Review and Critique; 3) Manuscript: A. Writing of the first draft, B. Review and Critique.

A.D.M.: 1A, 1B, 1C, 2A, 2B, 3A, 3B

I.D.: 2C, 3B

O.‐B.T.: 1A, 1B, 1C, 3B

J.P.L.: 1A, 1B, 1C, 3B

C.E.C.: 1A, 1B, 1C, 2C, 3B

## Financial disclosures for previous 12 months

A.D.M. has received funding support from Parkinson's UK, NHS Grampian Endowments, the Wellcome Trust, the University of Aberdeen, and the Academy of Medical Sciences. O.‐B.T. has given lectures for several pharmaceutical companies and is on the editorial board of *Acta Neurologica Scandinavica*. J.P.L. was associate editor of the *Journal of Parkinson's Disease* 2012 to 2016 and has received research funding from the Western Norway Health Trust, project support, 2013 to 2018. C.E.C. has received research funding from Parkinson's UK and NHS Grampian Endowments. I.D. reports no disclosures.

## Supporting information

Additional Supporting Information may be found in the online version of this article at the publisher's website

Supplementary Information 1

Supplementary Information 2

Supplementary Information 3
