# Supplementary figures and images for: Development and validation of prognostic survival models in newly diagnosed Parkinson's disease
Source: Mov Disord. 2017 Oct 4;33(1):108–16. doi: 10.1002/mds.27177 (PMC5813201; doi:10.1002/mds.27177)

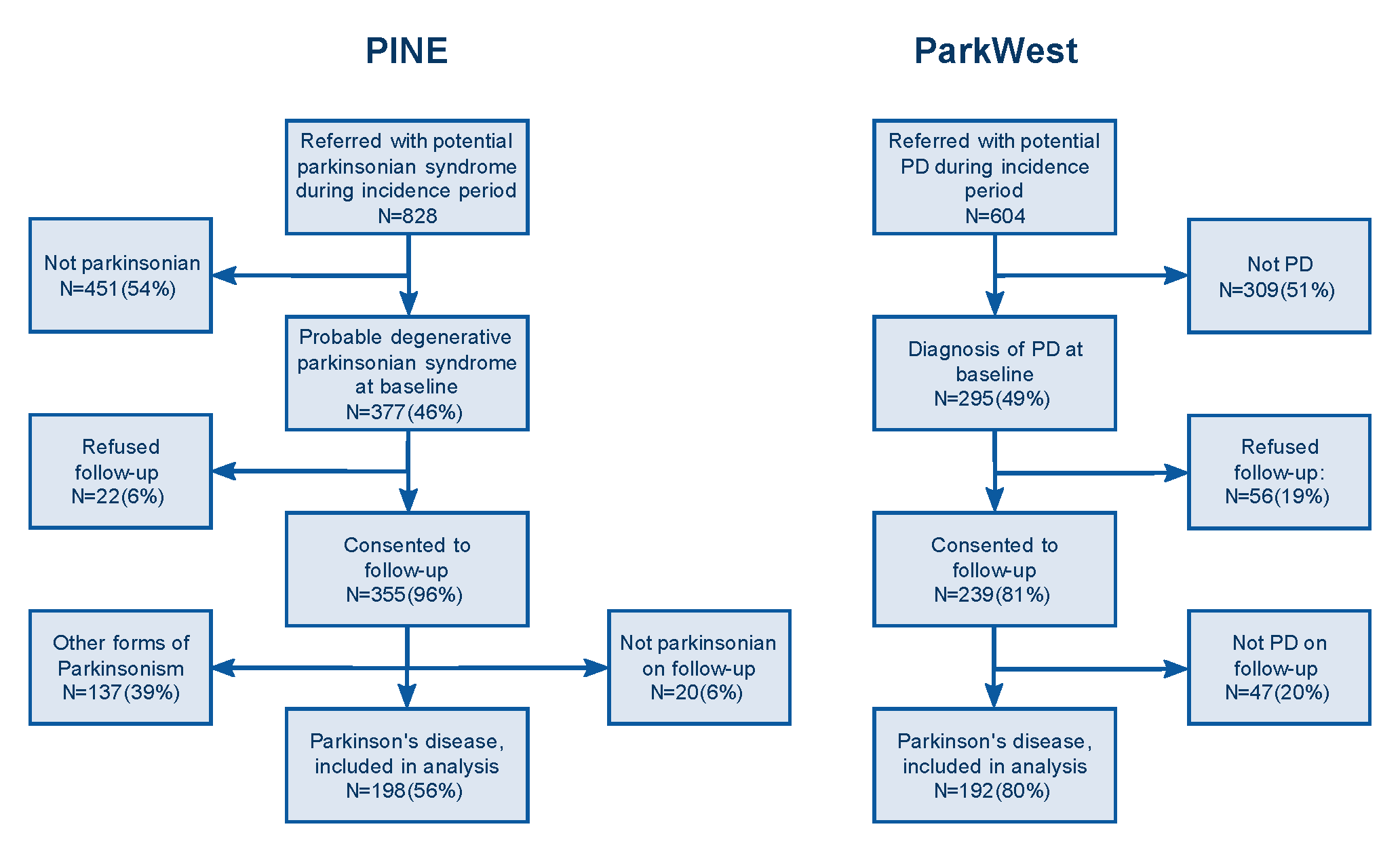

Supplement: Supplementary file 2 — Supplementary Information 2 [file MDS-33-108-s002.tiff]
